# Selecting Feature Subsets Based on SVM-RFE and the Overlapping Ratio with Applications in Bioinformatics

**DOI:** 10.3390/molecules23010052

**Published:** 2017-12-26

**Authors:** Xiaohui Lin, Chao Li, Yanhui Zhang, Benzhe Su, Meng Fan, Hai Wei

**Affiliations:** School of Computer Science and Technology, Dalian University of Technology, Dalian 116024, China; lizimu@mail.dlut.edu.cn (C.L.); yanhui.zhang.123@foxmail.com (Y.Z.); benzhe.su.123@foxmail.com (B.S.); mengfan2351@163.com (M.F.); weihai_dlut@163.com (H.W.)

**Keywords:** SVM-RFE, overlapping degree, feature selection

## Abstract

Feature selection is an important topic in bioinformatics. Defining informative features from complex high dimensional biological data is critical in disease study, drug development, etc. Support vector machine-recursive feature elimination (SVM-RFE) is an efficient feature selection technique that has shown its power in many applications. It ranks the features according to the recursive feature deletion sequence based on SVM. In this study, we propose a method, SVM-RFE-OA, which combines the classification accuracy rate and the average overlapping ratio of the samples to determine the number of features to be selected from the feature rank of SVM-RFE. Meanwhile, to measure the feature weights more accurately, we propose a modified SVM-RFE-OA (M-SVM-RFE-OA) algorithm that temporally screens out the samples lying in a heavy overlapping area in each iteration. The experiments on the eight public biological datasets show that the discriminative ability of the feature subset could be measured more accurately by combining the classification accuracy rate with the average overlapping degree of the samples compared with using the classification accuracy rate alone, and shielding the samples in the overlapping area made the calculation of the feature weights more stable and accurate. The methods proposed in this study can also be used with other RFE techniques to define potential biomarkers from big biological data.

## 1. Introduction

Feature selection is one of the main data analysis techniques in data mining, which has shown its power in many applications, such as insulator detection [1], medicine study [2], and environmental science [3]. Especially for big data analysis, how to define meaningful information is a key issue.

Along with the quick development of the high throughput techniques, genomics, metabolomics, and proteomics have been widely applied in disease study, drug research, etc. One characteristic of omics data is summarized in the expression ”high dimensions, small samples”, since omics data usually have a large number of features but few samples. As genomics data, metabolomics data and proteomics data usually contain many features, it has been critical to accurately measure the feature importance and select the most discriminative feature subset. Puthiyedth et al. [4] presented a combinatorial optimization approach for integrated feature selection and applied it to analyzing the data about prostate cancer. They have identified potential novel prostate cancer associated pathways and genes. Christin et al. [5] studied six feature selection methods, analyzed their performance for liquid chromatography-mass spectrometry based proteomics and metabolomics biomarker discovery. Zou et al. [6,7] presented the sequence based feature selection technique and dimensionality reduction strategy to realize the prediction of protein. Lin et al. [8] studied the feature selection method based on the overlapping area and defined the discriminative features of liver disease from the metabolomics dataset. 

Support vector machine (SVM) [9] is a popular and efficient classification technique and has been widely applied in many fields such as biological data processing [10]. SVM-recursive feature elimination (SVM-RFE) [11] is a feature selection algorithm based on SVM. While the SVM learning model is built, the weights of the features are also computed. SVM-RFE iteratively removes the features with the lowest weights. The removing sequence of the features represents the feature importance ranking [11,12]. SVM-RFE has been adopted in many applications, such as signal processing [13], genomics [11,12], proteomics [14] and metabolomics [15,16], due to its superiority. Also, many studies have been done on it to get a more powerful performance. Tang et al. [17] proposed a two-stage SVM-RFE. In the first stage, multiple SVM-RFEs with different parameters were applied to remove the noise and non-informative data; in the second stage, the final feature subset was selected by a fine SVM-RFE. Li et al. [18] combined SVM-RFE with the T-statistic to define the genes associated with CRC development or metastasis. mRMR-SVM [19] tries to select an important and non-redundant feature subset by means of SVM-RFE and mRMR. R-SVM [20] is also a recursive feature selection method based on SVM, which combines SVM weights and class means to evaluate feature discriminative abilities. There are also some studies on determining how many features with the low weights are removed in each iteration of SVM-RFE [21,22]. 

Basically, SVM-RFE ranks the features according to the feature deletion order during the iterations. The top ranked features which are removed in the last iteration of SVM-RFE are the most important, while the bottom ranked ones are the least informative and removed in the first iteration. For a specific application, it is not enough to obtain a feature importance ranking; it needs to determine how many top ranked features (such as genes and metabolites) should be selected. Thus, based on the selected features, we can study the disease phenotype and disease mechanism. In some studies, the top ranked features were selected according to a predetermined number [23,24]. In other studies, the top features that can induce a classifier with a “best” classification accuracy rate were selected [11,16].

It is not practical to specify the number of features to be selected in advance in some applications. However, it is well known that the feature subset selected should have a powerful discriminative ability. If a feature subset has a powerful discriminative ability, then the classifier based on it usually has a high prediction accuracy rate, and the different sample groups on the selected subspace should show different distributions with little overlapping areas. Hence, this study proposes a method, SVM-RFE-OA, which determines the number of features to be selected from the feature rank of SVM-RFE by combining the classification accuracy rate and the average overlapping ratio of the samples together. In addition, to weigh the features more accurately, this study also proposes a modified SVM-RFE-OA (M-SVM-RFE-OA) algorithm, which temporally screens out the samples lying in a heavily overlapping area in each iteration. The experiments on the eight public biological datasets show the validation of the two techniques proposed.

## 2. Methods

### 2.1. Overlapping Degree

Let *X* = {$x_{1}$, $x_{2}$, … $x_{n}$} be the dataset containing *n* samples, *C* be the class label set, *Label*($x_{i}$) ∈ *C* be the class label of sample $x_{i}$ ∈ *X*. For a sample $x_{i}$ ∈ *X*, the number of its neighbor samples that do not belong to the same class as $x_{i}$ reflects whether it lies in an overlapping area [25,26]. If most of its neighbors do not belong to the same class as $x_{i}$, then $x_{i}$ heavily mixes with the heterogeneous samples and locates in an overlapping area. Here we define *r*($x_{i}$) to represent the overlapping degree of sample $x_{i}$ based on the ratio of the heterogeneous samples in its neighborhood as follows:

(1) $$r(x_{i})=\frac{\mathrm{Difflabel}(x_{i})}{k}-OR(x_{i})$$

where *Difflabel*($x_{i}$) = {*x* | *x* ∈ *kNN*($x_{i}$) && *Label*(*x*) ≠ *Label*($x_{i}$)}, *kNN*($x_{i}$) is the set of the *k* nearest samples of $x_{i}$ [25,26], *OR*($x_{i}$) = {*x* | *x* ∈ *X*, *Label*(*x*) ≠ *Label*($x_{i}$)}/*n*. *Difflabel*($x_{i}$)/*k* is the heterogeneous sample ratio in the neighborhoods of $x_{i}$, and *OR*($x_{i}$) is the heterogeneous sample ratio in the training data.

*r*($x_{i}$) > 0 means that, in the neighbor area of sample $x_{i}$, the ratio of the samples belonging to the different class as $x_{i}$ is larger than the ratio of the samples belonging to the different class as $x_{i}$ in the whole training data, there are too many heterogeneous samples in the neighbor area of sample $x_{i}$.

To measure the overlapping degrees of the samples without bias, *r*($x_{i}$) is normalized as follows:

(2) $$Nr(x_{i})=\frac{r(x_{i})}{OR(x_{i})}$$

Therefore, *Nr*(*x*) represents the degree that sample *x* mixes with heterogeneous samples, and the average *Nr*(*x*) of all samples in the dataset reflects the mixing degree of the different class samples on the current subspace. If different classes show different distributions on the current feature subspace, then there is a clear separation among different classes, and the average *Nr*(*x*) of all samples is small. If different classes show almost the same distribution, they mix together on the subspace, and the average *Nr*(*x*) of all samples is large. Hence, the average *Nr*(*x*) can express how much discriminative information the current feature subset contains.

### 2.2. Feature Selection Based on SVM-RFE, the Overlapping Degree, and the Accuracy Rate

SVM-RFE [11,12] is a backward feature deletion method. At first, the current feature subset *F* contains all the input features. In each loop, an SVM learning model is built based on the current feature subset *F*, the weight (|*w*|) of each feature in *F* is calculated according to the support vectors on the hyper-plane of the SVM classifier. The features are then ranked based on |*w*|, and the bottom ranked features are removed from *F*. This procedure is repeated until *F* is empty. The feature removing sequence represents the feature importance rank [11,12]. The later the features are removed from *F*, the more important the features are. The top ranked features are those that are removed from *F* in the last iteration of SVM-RFE.

Thus, we can obtain a feature rank via SVM-RFE. However, for a certain data analysis, how many top ranked features should be selected from the feature rank of SVM-RFE is still to be considered. In some cases, the number of features to be selected is decided according to prior knowledge or is simply decided subjectively [23]. In other cases, the “optimal” feature subset is kept during the iteration as the final selected feature subset [11,16]. That is, in each iteration, the accuracy rate of the SVM learning model and the feature weights are calculated, and the features having the smallest weights are removed from *F*. When the procedure terminates, the feature subset corresponding to the maximal accuracy rate is kept as the final selected feature subset [11,16].

In biological data analysis, defining the most informative features (such as genes and metabolites) from the large complex data is of great importance to disease diagnosis and drug study. SVM-RFE is very efficient in analysis of large complex data. However, it is quite difficult to predetermine how many top ranked features should be selected from the feature rank of SVM-RFE. The classification accuracy rate of *d*-fold cross validation on the training dataset can be applied to determine which feature subset is selected during the backward feature deletion, i.e., the number of the selected features is determined by the classification accuracy rate on the training dataset. However, classification accuracy reflects the discriminative ability of the feature subset based on the classifier, and the distribution of the samples can also reflect the discriminative ability of the feature subset. If different class samples mix together on the current subspace, the overlapping degree of the samples is large, and the subspace has little discriminative information. Both the classification accuracy and the overlapping degree of the samples can tell us how much discriminative information the feature subset has. They evaluate the feature subset from two different aspects, respectively. The discriminative ability of the feature subset could be evaluated more comprehensively by combining these two terms. Hence, we propose SVM-RFE-OA (see Algorithm 1 SVM-RFE-OA), which measures the feature subset during the iterations of SVM-RFE by integrating the average overlapping degree of samples and the classification accuracy rate, and selects a feature subset that has a large accuracy rate and a small overlapping degree. In each iteration, SVM-RFE-OA calculates the average accuracy rate (*T_c_acc*) of *d*-fold cross validation and the average *Nr*(*x*) (*T_c_oa*) of all the samples in the training data, and the feature subset having the largest “*T_c_acc* − *T_c_oa*” is kept as the final selected feature subset.
**Algorithm 1** SVM-RFE-OAInput: training dataset *X*, *t*.Output: selected feature subset *FS*.Begin  *c_acc* = 0;  *c_oa* = ∞;  *F* = {all input features};  While (|*F*| > 0) Do   Construct an SVM based on *X* and *F*;   *T_c_acc* = *d*-fold cross validation accuracy rate of SVM;   *T_c_oa* = average *Nr*(*x*) of the samples in *X* based on *F*;   Rank the features in *F* by |*w*| in descending order;   If  *T_c_acc* − *T_c_oa* > *c_acc* − *c_oa* Then     *c_acc* = *T_c_acc*;     *c_oa* = *T_c_oa*;      *FS* = *F*;   Endif;      *F* = *F*− {*t* × |*F*| bottom ranked features in *F*};  Endwhile;  Return *FS*;End.

*t* (0 < *t* < 100%) is the filter factor. In each iteration of SVM-RFE, *t* × |*F*| bottom ranked features are removed from the current feature subset *F*.

### 2.3. Modified-SVM-RFE-OA

In the calculation of feature weights, only the samples on the hyper-plane of the SVM learning model are considered [11,12]. The hyper-plane is constructed based on the training samples and the current subspace. The quality of the training data can affect the hyper-plane construction and the computation of feature weights. If different group samples mix heavily on the subspace, overfitting may occur, which can induce the bias of the calculation of feature weights. Therefore, to get a more accurate calculation of the feature weights, we propose a modified algorithm based on SVM-RFE-OA (M-SVM-RFE-OA), which temporally screens out the samples lying in a heavy overlapping area in each iteration (see Algorithm 2 M-SVM-RFE-OA). That is, (1) in each iteration, *Nr*(*x*) of each sample in the training data is calculated based on the current subspace *F*; (2) the samples with *Nr*(*x*) > 0 are temporarily set aside and are not used in SVM training in this iteration. At most, one-third of the samples in each class in the training data are screened out to make sure that there are enough samples kept for the training. Since the samples in the heavy overlapping area are shielded in the training procedure, there is little chance that overfitting occurs, and the bias becomes small.
**Algorithm 2**
M-SVM-RFE-OAInput: training dataset *X*, *t*.Output: selected feature subset *FS*.Begin  *c_acc* = 0;   *c_oa* = ∞;  *F* = {all input features};  While (|*F*| > 0) Do   Calculate *Nr*(*x*) for each *x* ∈ *X* based on *F*;   *X_t_ = X*;   For each *c* ∈ *C* Do    *X_c_* = {*x* | *x* ∈ *X*, *Label*(*x*) = *c* and *Nr*(*x*) > 0};    *θ* = |{*x* | *x* ∈ *X*, *Label*(*x*) = *c*}|;    If |*X_c_*| > *θ*/3 Then     Rank the samples in *X_c_* based on *Nr*(*x*) in descending order;     *X_t_* = *X_t_* − {*θ*/3 top ranked samples in *X_c_*};    Else      *X_t_ = X_t_ − X_c_*;    End if;   End for;     Construct an SVM based on *X_t_* and *F*;     *T_c_acc* = accuracy rate of SVM;     *T_c_oa* = average *Nr*(*x*) of the samples in *X_t_* based on *F*;     If   *T_c_acc − T_c_oa* > *c_acc − c_oa* Then     *c_acc = T_c_acc*;     *c_oa = T_c_oa*;     *FS = F;*    End if;    Rank the features in *F* by |*w*| in descending order;    *F* = *F −* {*t* × |*F*| bottom ranked features in *F*};  End while;  Return *FS*;End

## 3. Results and Discussion

To show the performance of the two techniques proposed, SVM-RFE-OA and M-SVM-RFE-OA were compared with SVM-RFE where the selected feature subset was determined by the classification accuracy rate. Eight public biological datasets were used in the comparison of the three algorithms, where Breast2, Colon, Lymphoma, Prostate, Brain_data, Srbct are from http://ligarto.org/rdiaz/Papers/rfVS/randomForestVarSel.html, and the last two datasets are from www.gems-system.org. Breast2 [27,28] contains 77 samples, including 33 samples that developed distant metastases within 5 years and 44 samples that remained disease-free for over 5 years. The Colon [27,29] dataset includes 40 tumors samples and 22 normal colon tissues samples with 2000 genes by Affymetrix technology. The DLBCL_GEMS [30] dataset includes 58 diffuse large B-cell lymphomas (DLBCL) samples and 19 follicular lymphomas samples. The Lymphoma [27,31] dataset contains the most prevalent adult lymphoid malignancies. The total sample size is 62, including 42 samples of diffuse large B-cell lymphoma, 9 follicular lymphoma samples, and 11 chronic lymphocytic leukemia samples. Prostate [27,32] contains 52 prostate tumors samples and 50 non-tumor prostate samples. Brain_data [27,33] contains 42 samples, which include 5 different tumors of the central nervous system, 10 medulloblastomas samples, 10 malignant gliomas samples, 10 atypical teratoid/rhabdoid tumors (AT/RTs) samples, 8 primitive neuro-ectodermal tumors (PNETs) samples, and 4 human cerebella samples. The Leukemia2_GEMS [30] dataset contains 24 acute lymphoblastic leukemia (ALL) samples, 28 acute myeloid leukemia (AML) samples, and 20 mixed-lineage leukemia (MLL) samples. The Srbct [27,34] dataset, named the small, round blue cell tumors of childhood, includes 23 neuroblastoma (NB) samples, 20 rhabdomyosarcoma (RMS) samples, 12 non-Hodgkin lymphoma (NHL) samples, and 8 the Ewing family of tumors (EWS) samples. Table 1 provides detailed information of the eight datasets. Four of them are binary problems.

SVM-RFE, SVM-RFE-OA, and M-SVM-RFE-OA were implemented in C++. SVM was obtained from http://www.csie.ntu. edu.tw/~cjlin/libsvm/. Linear kernel was adopted in the SVM, and *t* was set to 5%. In SVM-RFE-OA and M-SVM-RFE-OA, *k* was set to 9. Five-fold cross validation was run 50 times for each method. The average classification accuracy rates and the average standard deviations are given in Table 2. For the four binary datasets, sensitivities and specificities are given in Table 3 and Table 4, respectively. In the tables, the bold numbers represent the largest value in a dataset among the three methods.

First, we compared SVM-RFE-OA with SVM-RFE to examine the performance of SVM-RFE-OA. Table 2 shows that SVM-RFE-OA outperforms SVM-RFE for seven of the eight biological datasets in classification accuracy rate. The accuracy rate of SVM-RFE-OA is higher than that of SVM-RFE by 8.95% for Brain_data. Only for Breast2 is the average accuracy rate of SVM-RFE-OA lower than that of SVM-RFE (by 0.83%). The sensitivities and specificities (see Table 3 and Table 4) for the four binary problems also show the superiority of SVM-RFE-OA over SVM-RFE. The sensitivities of SVM-RFE-OA are higher than those of SVM-RFE for three of the four binary datasets, and its specificities are higher than those of SVM-RFE for three of the four datasets, too. Hence, the discriminative ability of the feature subset could be measured more accurately by combining the classification accuracy rate with the average overlapping degree of samples than by using the classification accuracy rate alone. The classification accuracy reflects feature distinguishing ability via the classification model, while the average overlapping degree of the samples represents the discriminative information that the feature subset contains by means of the sample distribution. Combining the two criteria induces a more comprehensive measurement of the feature subset. This technique can be used in other RFE analyses to determine the final selected feature subset.

Secondly, we compared M-SVM-RFE-OA with SVM-RFE-OA, thereby examining the performance of temporally screening out the poor samples lying in an overlapping area. Both M-SVM-RFE-OA and SVM-RFE-OA combine the classification accuracy rate and the average overlapping degree to calculate the discriminative ability of the feature subset and determine the number of top ranked features to be selected. To measure the feature importance more accurately, M-SVM-RFE-OA temporarily shields the samples in the overlapping area in each iteration. The comparison between M-SVM-RFE-OA and SVM-RFE-OA shows that temporarily screening out the samples mixed with heterogeneous samples in each iteration benefits the calculation of feature weights. Table 2 clearly shows that M-SVM-RFE-OA outperforms SVM-RFE-OA for seven of the eight datasets in terms of the accuracy rate. Table 3 and Table 4 also represent the superiority of M-SVM-RFE-OA over SVM-RFE-OA in sensitivity and specificity. Therefore, we have that the quality of the training data influences the construction of the SVM model and the calculation of feature weights. M-SVM-RFE-OA produces a more accurate calculation of the feature weights by temporally screening out the samples with high overlapping ratios in each iteration, finally obtaining a more powerful feature subset.

The comparisons between SVM-RFE and SVM-RFE-OA and between SVM-RFE-OA and M-SVM-RFE-OA validate the two techniques proposed in this study. Finally, it can be seen that M-SVM-RFE-OA outperforms SVM-RFE for all eight datasets in terms of accuracy rate and outperforms SVM-RFE for all the four binary datasets in terms of sensitivity and specificity. Especially for Brain_data, the accuracy rate of M-SVM-RFE-OA is higher than that of SVM-RFE by 10.2%.

Meanwhile, SVM-RFE-OA and M-SVM-RFE-OA are more stable than SVM-RFE. The standard deviations of M-SVM-RFE-OA on accuracy rate, sensitivity, and specificity are lower than those of SVM-RFE in most cases. Hence, from two different aspects, the classification accuracy rate and the average overlapping degree of samples, which reflects the sample distribution on the feature subspace (top ranked feature subset), we can obtain a more comprehensive measurement of the feature subset. Further, temporally shielding the samples with high overlapping ratios in each iteration could make the computation of feature importance more accurate.

Table 5 gives the average number of features selected in five-fold cross validation run 50 times for each method. It can be seen that the average number of features selected by SVM-RFE is less than those selected by SVM-RFE-OA and M-SVM-RFE-OA. However, the classification accuracy rates of SVM-RFE-OA and M-SVM-RFE-OA are higher than those of SVM-RFE, and the standard deviations of SVM-RFE-OA and M-SVM-RFE-OA are lower than those of SVM-RFE (see Table 2). For the Lymphoma dataset, the average number of selected features by SVM-RFE is 3.48, while SVM-RFE-OA and M-SVM-RFE-OA increase the classification accuracy rate 0.93% and 1.49% by 1.59 and 1.62 more features, respectively. Although the average numbers of features selected by SVM-RFE-OA and M-SVM-RFE-OA are larger than those by SVM-RFE, the two new methods are much more efficient and stable than SVM-RFE.

## 4. Conclusions

In systems biology, it is very significant to select the most meaningful features from large complex genomics, metabolomics, and proteomics data, which could help in classifying different disease samples, studying disease mechanisms, and developing new drugs. This paper proposes two techniques of selecting discriminative feature subsets based on SVM-RFE. One is measuring the feature subset by combining the classification accuracy rate with the average overlapping degree of samples, and the other is temporally screening out the samples in a heavily overlapping area in each loop of the SVM-RFE. Experiments on eight public biological datasets show the validation of these techniques and prove that filtering out the samples that lie in the heavily overlapping area could make the measurement of feature weights more accurate.

## Figures and Tables

**Table 1:** Data description.

Datasets	No. of Samples	No. of Features	No. of Classes
Breast2 [27,28]	77	4869	2
Colon [27,29]	62	2000	2
DLBCL_GEMS [30]	77	5469	2
Lymphoma [27,31]	62	4026	3
Prostate [27,32]	102	6033	2
Brain_data [27,33]	42	5597	5
Leukemia2_GEMS [30]	72	11225	3
Srbct [27,34]	63	2308	4

**Table 2:** Comparison in accuracy (%).

Datasets	SVM-RFE	SVM-RFE-OA	M-SVM-RFE-OA
Breast2	61.96 ± 4.57	61.13 *±* 4.17	**65.19 ± 3.77**
Colon	80.39 ± 3.99	83.92 ± 2.97	**88.61 ± 1.44**
DLBCL_GEMS	89.09 ± 4.35	**94.29 ± 2.25**	93.69 ± 2.97
Lymphoma	94.14 ± 2.63	95.07 ± 2.25	**95.63 ± 2.38**
Prostate	89.46 ± 2.14	91.84 ± 1.82	**92.24 ± 1.56**
Brain_data	71.78 ± 5.09	80.63 ± 4.58	**81.98 ± 3.21**
Leukemia2_GEMS	89.83 ± 2.80	94.39 ± 2.28	**94.69 ± 2.03**
Srbct	95.21 ± 2.79	98.43 ± 1.45	**98.59 ± 1.44**

Bold: the largest value in a dataset among the three methods.

**Table 3:** Comparison in sensitivity (%).

Datasets	SVM-RFE	SVM-RFE-OA	M-SVM-RFE-OA
Breast2	66.95 ± 4.99	66.45 ± 4.94	**68.27 ± 5.07**
Colon	86.35 ± 3.40	88.65 ± 2.27	**90.15 ± 1.63**
DLBCL_GEMS	81.16 ± 8.97	**88.32 ± 5.55**	88.32 ± 6.49
Prostate	89.38 ± 2.67	**91.19 ± 2.17**	90.23 ± 2.01

Bold: the largest value in a dataset among the three methods.

**Table 4:** Comparison in specificity (%).

Datasets	SVM-RFE	SVM-RFE-OA	M-SVM-RFE-OA
Breast2	55.27 ± 7.23	53.94 ± 7.55	**61.03 ± 6.27**
Colon	69.45 ± 9.72	75.27 ± 6.76	**85.82 ± 2.70**
DLBCL_GEMS	91.72 ± 4.14	**96.24 ± 2.43**	95.45 ± 2.78
Prostate	89.52 ± 3.65	92.48 ± 2.84	**94.32 *±* 2.19**

Bold: the largest value in a dataset among the three methods.

**Table 5:** The average number of features selected.

Datasets	SVM-RFE	SVM-RFE-OA	M-SVM-RFE-OA
Breast2	17.54	52.34	44.89
Colon	12.41	29.46	52.36
DLBCL_GEMS	7.62	39.54	34.50
Lymphoma	3.48	5.07	5.10
Prostate	12.73	60.16	57.50
Brain_data	12.94	48.15	121.68
Leukemia2_GEMS	9.61	78.49	73.99
Srbct	7.22	31.04	30.18
